# Targeted Delivery of CD34 Aptamer-Coupled Tocilizumab Microspheres for Effective Treatment of Thyroid-Associated Ophthalmopathy

**DOI:** 10.1167/iovs.66.11.57

**Published:** 2025-08-25

**Authors:** Yong Luo, Jiayang Yin, Jiamin Cao, Bingyu Xie, Feng Zhang, Sha Ouyang, Juan Zhou, Yao Tan, Wei Xiong

**Affiliations:** 1 Department of Ophthalmology, Third Xiangya Hospital, Central South University, Changsha, Hunan, People's Republic of China

**Keywords:** CD34 positive fibroblasts, Tocilizumab (Toc), PLGA microspheres, thyroid-associated ophthalmopathy (TAO), IL-6 receptor

## Abstract

**Purpose:**

Thyroid-associated ophthalmopathy (TAO) is a debilitating autoimmune disorder linked to Graves’ disease (GD) that is characterized by inflammation and remodeling of orbital tissues. This study focuses on the use of poly(lactic-co-glycolic) acid (PLGA)-based microspheres (MS) coupled with CD34-specific aptamers to enhance the targeted delivery of Tocilizumab (Toc), an IL-6 receptor monoclonal antibody, to CD34^+^ orbital fibroblasts, which is a critical cell type implicated in TAO.

**Methods:**

The flow cytometry and aptamer-mediated pull-down assays were applied to detect the affinity of CD34 aptamers (Apts) for CD34^+^ orbital fibroblasts. The CD34 Apt-modified Toc-loaded MS (Toc-MS-CD34 Apt) were characterized by scanning electron microscopy (SEM), transmission electron microscopy (TEM), Fourier transform infrared spectroscopy (FT-IR), and agarose gel electrophoresis. The CCK-8 assay kit was used to examine cell viability. The EdU assay was used to assess cell proliferation. The scratch wound healing assay was applied to detect cell migration.

**Results:**

The affinity of CD34 aptamers for CD34^+^ orbital fibroblasts was confirmed, demonstrating high specificity and binding strength. The Toc-MS-CD34 Apt exhibited size uniformity and successful aptamer conjugation. In vitro, studies showed that Toc-MS-CD34 Apt effectively inhibited the viability, proliferation and cell activation, extracellular matrix (ECM) protein expression, and migration of TGF-β1-stimulated CD34^+^ orbital fibroblasts. In vivo, a TAO mouse model treated with anti-mouse IL-6R microspheres (anti-mmu-IL-6R-MS) and CD34 aptamer-modified anti-mmu-IL-6R microspheres (anti-mmu-IL-6R-MS-CD34 Apt) demonstrated significant reductions in tissue inflammation, fibrosis, and ECM protein levels, with notable inhibition of the STAT3 signaling pathway.

**Conclusions:**

These findings highlight the potential of CD34 aptamer-coupled Toc microspheres as a targeted therapy for TAO, providing a comprehensive strategy that addresses local manifestations of the disease.

Thyroid-associated ophthalmopathy (TAO) is a debilitating autoimmune disorder linked to Graves’ disease (GD) that often leads to inflammation and remodeling of orbital tissues.^1^ In GD, thyroid-stimulating immunoglobulin (TSI) targets the thyroid-stimulating hormone receptor (TSHR), triggering thyroid dysfunction.^2^^–^^4^ TSHR is also present in orbital connective tissue and fibroblasts, where its activation promotes pro-inflammatory cytokine production, such as IL-6, which exacerbates inflammation and amplifies TSHR expression, creating a vicious cycle.^5^^,^^6^ Despite strides made in the comprehension of TAO pathophysiology, managing the disease remains challenging, given the limited effectiveness of current treatments. Tocilizumab (Toc) is a monoclonal antibody targeting IL-6 receptors. It has shown effectiveness in patients with TAO who are unresponsive to steroids^7^; however, its therapeutic efficacy is hindered by challenges in targeted delivery, highlighting the need for improved treatment strategies.

CD34^+^ fibroblasts play a key role in TAO pathogenesis.^8^^,^^9^ In GD, the number of circulating CD34^+^ fibrocytes is significantly elevated, particularly in patients with active TAO.^10^ These fibrocytes infiltrate the orbit and differentiate into CD34^+^ orbital fibroblasts, which are notably absent in healthy tissues, highlighting their specific role in TAO pathology.^10^ This subset of CD34^+^ orbital fibroblasts, characterized by high TSHR expression, exhibits enhanced pro-inflammatory activity, producing cytokines such as IL-6 and TNF-α in response to thyroid-stimulating hormones (TSHs) and TSIs.^11^^,^^12^ This pro-inflammatory activity contributes to the tissue remodeling and fibrotic characteristics of TAO. Therefore, targeting CD34^+^ orbital fibroblasts represents a promising therapeutic strategy. The use of CD34-specific agents, such as aptamers (Apts), to deliver Toc directly to these cells may enhance treatment efficacy by reducing inflammation and fibrosis, ultimately improving clinical outcomes in patients with TAO.

Given the challenges with conventional TAO treatments, improving targeted drug delivery using poly(lactic-co-glycolic) acid (PLGA)-based microspheres coupled with Apts was the focus of our study. PLGA is a US Food and Drug Administration (FDA)-approved biomaterial that is known for its biocompatibility, non-toxicity, and controlled degradation, making it an ideal candidate for drug encapsulation.^13^^,^^14^ Apts are single-stranded DNA or RNA molecules that offer high selectivity and specificity toward their targets, providing singular advantages over conventional antibodies.^15^^,^^16^ They have been used to capture various target molecules, including cells, proteins, and small molecules.^17^^–^^19^ In TAO, targeting CD34^+^ orbital fibroblasts with Apts presents a promising therapeutic approach, given their critical role in disease pathology.^8^^–^^12^ By coupling Apts with PLGA microspheres, we aim to achieve sustained and controlled delivery of Toc directly to CD34^+^ orbital fibroblasts, thereby enhancing therapeutic efficacy, minimizing systemic side effects, and improving clinical outcomes in patients with TAO by attenuating inflammation and fibrosis.

Apts targeting CD34^+^ orbital fibroblasts in this study were screened using the Systematic Evolution of Ligands by Exponential Enrichment (SELEX) technology, Toc-loaded PLGA microspheres (MS; Toc-MS) and CD34^+^ specific aptamers coupled Toc-MS (Toc-MS-CD34 Apt) were prepared and characterized, and their effects on TGF-β-stimulated orbital fibroblasts were investigated. Additionally, mouse anti-IL-6R loaded microspheres ((anti-mmu-IL-6R)-MS) and CD34^+^ specific Apts coupled Anti-IL-6R-MS ((anti-mmu-IL-6R)-MS-CD34 Apt) was prepared and characterized and then tested in a TAO mouse model. Collectively, these approaches aim to enhance the targeted delivery and therapeutic efficacy of treatments for TAO, potentially improving clinical outcomes by mitigating fibrosis and inflammation in orbital fibroblasts.

## Materials and Methods

### Collection of Clinical Samples

Control samples of orbital tissues were collected from healthy volunteers (*n* = 6) who did not have any inflammatory or thyroid diseases and underwent ptosis correction surgery. Orbital tissue samples were harvested from individuals with TAO (*n* = 6; inactive disease stage) during orbital decompression surgery. Half of the samples were subjected to histological examination, whereas the other half of the samples were used for primary fibroblast isolation. Each recruited participant signed an informed consent form in accordance with the standards outlined in the Declaration of Helsinki. The study received approval from the Medicine Ethics Committee at the Third Xiangya Hospital.

### Histopathological Examination

The thyroid tissue and orbital tissue underwent histological investigations. The histological investigation was performed using established assays.^20^^,^^21^ The tissue samples were preserved in a solution of 10% buffered formalin, and then enclosed in paraffin and analyzed using hematoxylin and eosin (H&E) or Masson on consecutive slices. A microscope (Olympus, Kyoto, Japan) was used to analyze the slices.

### Isolation of Orbital Fibroblasts

Orbital fat tissues were harvested from patients with TAO undergoing orbital decompression surgery. The tissues were washed with 5 mL of PBS buffer to remove surface blood vessels. After washing, the tissues were soaked in 1 mL of high-glucose basal medium and finely minced into fragments approximately 1 mm in diameter using sterilized ophthalmic scissors. The homogenized tissue was then transferred to a 10 mL centrifuge tube and mixed with an appropriate amount of type II collagenase. The tube was incubated at 37°C for 1 hour, followed by the addition of 1 mL of serum to terminate the digestion. The tissue homogenate was filtered through a sieve and washed with PBS buffer. The filtered suspension was centrifuged, and the supernatant was removed. The red blood cells were lysed using a red blood cell lysis solution, followed by another centrifugation and a single wash with 1 mL of PBS buffer. The resulting cell suspension was transferred to a culture dish and cultured in a complete medium for 4 hours. The non-adherent cells were washed with PBS. Finally, the adherent cells were identified as orbital fibroblasts by immunofluorescent staining. Orbital fibroblasts were positively marked with vimentin and negatively marked with desmin (DES), KRT17, myoglobin (MB), and S100B.^22^

### Immunofluorescent Staining 

Orbital fibroblasts (1 × 10^4^) were placed in a culture plate and cultured overnight. The orbital fibroblasts were washed with PBS and treated with 4% paraformaldehyde for 20 minutes. Afterward, they were blocked and permeabilized using a mixture of 3% BSA, 0.3%, and Triton X-100 in PBS for 1 hour at room temperature (RT). The orbital fibroblasts subsequently underwent overnight incubation with the primary antibodies against vimentin (10366-1-AP; Proteintech, Wuhan, China), α-SMA (14395-1-AP; Proteintech), collagen I (14695-1-AP; Proteintech), keratin 17 (KRT17; 17516-1-AP; Proteintech), DES (16520-1-AP; Proteintech), MB (16048-1-AP; Proteintech), and S100B (15146-1-AP, Proteintech) followed by incubation with the secondary antibody labeled with either Alexa Fluor 488 or Alexa Fluor 594 (Thermo Fisher Scientific, Waltham, MA, USA) for a duration of 2 hours at RT. Following counterstaining with 4′,6-diamidino-2-phenylindole (DAPI), the orbital fibroblasts were seen and captured using a fluorescence microscope (Olympus).^23^

### Screening of CD34^+^ Orbital Fibroblasts

One culture dish of primary orbital fibroblasts from patients with TAO was collected and incubated with Fc receptor blocking solution (Biolegend, San Diego, CA, USA) for 5 minutes, and FITC labeled CD34 flow cytometry antibodies (343504; Biolegend) were added. The cells were incubated with the antibodies for 20 minutes in light-deprived conditions on ice and then washed twice with PBS buffer. The primary orbital fibroblasts were then sorted into CD34^+^ and CD34^–^ subpopulations using a flow cytometric cell sorter (BD Biosciences, San Jose, CA, USA). Both cell populations were expanded in culture, and the expression of CD34 was re-evaluated using flow cytometry to confirm that the sorted cells were indeed primary CD34^+^ and CD34^–^ orbital fibroblasts.^24^

### Screening of CD34^+^ Recognizing Aptamers 

The aptamers that exhibit selective binding to CD34^+^ orbital fibroblasts were chosen by using SELEX technology based on previous research.^25^ An 80-base ssDNA initial randomized library containing 40 random oligonucleotides was designed as follows: 5′-CAGCACCGTCAACTGAAT (40 randomized nucleotides) GTGATGCGATGGAGATGT-3′, and the FAM-labeled ssDNA library was generated by PCR (Forward primers = 5′-FAM-CAGCACCGTCAACTGAAT-3′; Reverse primer = 5′-Biotin- ACATCTCCATCGCATCAC-3′), and purified by the biotin-streptavidin magnetic bead system. Before each incubation, the ssDNA library was heated at 95°C and chilled on ice to induce a specific conformational change. After incubating with CD34^+^ orbital fibroblasts in binding buffer (4.5 mg/mL D-glucose, 5 mM MgCl_2_, 1 mg/mL BSA, and 1 mg/mL yeast tRNA in DPBS) for 1 hour at 4°C in the dark, wash buffer (4.5 mg/mL D-glucose and 5 mM MgCl_2_ in DPBS) was used to wash away the unbound ssDNA. The CD34^+^ orbital fibroblasts contained ssDNA were further harvested and incubated at 95°C for 10 minutes to elute the ssDNA. The eluted ssDNA were amplified, purified, and then used for cell incubation in the next round of screening. The binding affinity of ssDNA from successive rounds of SELEX to CD34^+^ orbital fibroblasts (positive screening target) and CD34^–^ orbital cells (negative screening) was determined to identify high-affinity Apts. The final ssDNA library of the eighth round screening exhibiting the highest affinity was sequenced by Sangon Biotech Co., Ltd. The sequence of CD34 Apt is 5′- CAGCACCGTCAACTGAATCTCATTGCGGCGTCCGGCTGGACGTTATTGTTGGAACCGTGATGCGATGGAGATGT-3′. Finally, the biological software NUPACK was applied to analyze the primary and secondary structures of the Apt clusters.

### Aptamer Affinity Determination Using Flow Cytometry

To assess the affinity between CD34 Apt and various cells, FAM-labeled CD34 Apt samples (General Bio, Chuzhou, China) were prepared at different concentrations and incubated with primary TAO mouse orbital fibroblasts, primary TAO human orbital fibroblasts, HEK293 cells, and human adipose-derived mesenchymal stem cells (CD34^–^) at 4°C for 1 hour. The initial FAM-labeled library served as a negative control. After incubation, the cells were washed thrice with 500 µL of washing buffer and resuspended in 500 µL of binding buffer. The fluorescence intensity of each sample was measured using a flow cytometer. The fluorescence intensity of the cells themselves was subtracted, and the mean fluorescence intensity (MFI) was plotted against the Apt concentration. The data were modeled using the equation *Y* = (*B*_max _*X*)/(*KD* + *X*) to determine the binding affinity.^26^

### Aptamer-Mediated Pull-Down Assay

At 4°C, TAO orbital fibroblasts were suspended in a hypotonic buffer. The debris was washed thrice with a hypotonic solution and dissolved in a lysis buffer (5 mM MgCl_2_ and 2% Triton X-100) at 4°C for 30 minutes after 1000g centrifugation. After centrifugation at 5500g and 4°C, the BCA Protein Assay Reagent kit (Beyotime, Shanghai, China) measured supernatant protein. Samples with 100 µg protein were blocked with 20% FBS and 0.1 mg/mL Salmon Sperm DNA for 60 minutes. Biotin-labeled CD34 Apt (150 pmoL) and the initial library as control were incubated overnight at 4°C. Protein-Apt complexes were incubated with 20 µL streptavidin-coated sepharose beads (GE Healthcare, Uppsala, Sweden) at 4°C by rotation for 1 hour. Positive controls were untreated cell lysates, whereas negative controls were beads alone. To elute target-bound proteins, beads were washed 3 times with D-PBS and heated at 95°C for 10 minutes in 30 µL of 2 × SDS-PAGE sample buffer. The target proteins were detected by anti-CD34 antibody (AF5149; Affinity Bioscience, Changzhou, China) immunoblotting.

### Preparation of Tocilizumab-Loaded Microspheres 

Tocilizumab-loaded microspheres (Toc-MS) were prepared using a water-in-oil-in-water (W/O/W) double emulsion method.^27^ For MS synthesis, 4 mL PLGA (100 mg) dichloromethane solution was mixed with 200 µL of 0.05% w/v polyvinyl alcohol (PVA) water solution and sonicate for 2 minutes (Soinfier Sound, Shanghai, China) on ice to create the W/O solution. The W/O solution was subsequently added to 10 mL of 0.5% w/v PVA and sonicated for 2 minutes on ice to yield the W/O/W solution. After being stirred at RT with magnetic stirring (800 rpm) for 4 hours and centrifugation at 13,000 rpm for 20 minutes at 4°C, the collected MS were washed with distilled water and lyophilized. For Toc-MS synthesis, 0.5 mg Toc (MedChem Express, Monmouth Junction, NJ, USA) water solution was mixed with 200 µL 0.05% w/v PVA and then mixed with 4 mL PLGA (100 mg) dichloromethane solution for 2 minutes of ice bath ultrasound to yield the W/O solution. The W/O solution was subsequently added into 0.5% w/v PVA. The Toc-MS was obtained following the same procedure as mentioned above. For the in vivo study, the Toc was replaced by anti-mmu IL-6R (Bio X cell, Lebanon, NH, USA), and the remaining procedure is identical to yield anti-mmu-IL-6R-MS.

### Preparation of CD34 Aptamer-Coupled Tocilizumab Microspheres 

The Toc-MS (2.5 mg) were dissolved in 250 µL of MES buffer (pH 5.5) to a concentration of 10 µg/µL and incubated with 200 µL of 400 mmol/L EDC and 200 µL of 100 mmol/L NHS at RT with gentle shaking for 30 minutes to activate the particles. The NHS-activated MS was then covalently linked to NH2-modified CD34 Apts (1 µg/µL). The sample was continuously stirred at RT for 2 hours, washed thrice with DNase-RNase-free water, and the resulting Toc-MS-CD34 Apt suspension was stored at 4°C. For the in vivo study, Toc was replaced by anti-mmu IL-6R, and the remaining procedure is identical to yield anti-mmu-IL-6R-MS-CD34 Apt.

### Morphology Observation by Scanning Electron Microscopy and Transmission Electron Microscopy 

The external morphology of Toc-MS and Toc-MS-CD34Apt was analyzed using a Hitachi S-510 scanning electron microscope (Hitachi-High Technologies Europe GmbH, Krefeld, Germany). The samples were applied to conductive carbon tabs, dried under a laminar flow hood, sputter-coated with gold using an Edwards S150 sputter coater, and examined at an acceleration voltage of 5 kilovolts (kV) and a working distance of 12 mm. The internal morphology was observed by transmission electron microscopy (TEM; JEOL, JEM-F200, Tokyo, Japan). The MS sample was dropped onto a carbon-coated copper grid and air-dried. The samples were observed at 200 kV.

### Fourier Transform Infrared Spectroscopy 

Nicolet iS20 Fourier transform infrared spectroscopy (FTIR) Spectrometer (Thermo Fisher Scientific) was used to evaluate the surface modification of the microspheres.^28^ Spectra were collected in the range of 4000 cm^−1^ to 400 cm^−1^ and expressed as percent transmittance.

### CD34 Apt Distribution by Agarose Gel Electrophoresis

The distribution of CD34 Apt on the microsphere surface was evaluated using agarose gel electrophoresis.^29^ A 1% (w/v) agarose gel was prepared with 60 mL TAE buffer, 0.6g agarose, and 6 µL Gel-red stain. Samples of Toc-MS, CD34 Apt, and Toc-MS-CD34 Apt were loaded into the gel wells and electrophoresed at 220 volts (V) for 15 minutes. Images were captured using a gel imaging system (Tanon, Shanghai, China).

### Drug Loading and Encapsulation Efficiency

The Toc-loaded MS (1 mg of Toc-MS or Toc-MS-CD34 Apt) were dissolved in 50% acetone-PBS to dissolve PLGA. The concentration of Toc in each sample was measured using an ELISA kit (EL-1611-202, Zhonghao Biotech). Drug loading (DL) and encapsulation efficiency (EE) were calculated as follows:

$$\begin{array}{c} DL\;=\;\left(W_{e}/W_{m}\right)\;\times \;100\% \end{array}$$

$$\begin{array}{c} EE\;=\;\left(W_{e}/W_{0}\right)\;\times \;100\% \end{array}$$

Where *W_e_* is the mass of the drug encapsulated in the microspheres, *W_m_* represents the total mass of the drug-loaded microspheres, and *W*_0_ stands for the theoretical mass of the drug.

### In Vitro Drug Release

The samples of Toc-MS and Toc-MS-CD34 Apt (50 mg each) were suspended in 10 mL PBS and stirred continuously (300 rpm/min, 37°C). At specific time points, 50 µL of the suspension was collected. The Toc concentration in the supernatant was measured using an ELISA kit (EL-1611-202; Zhonghao Biotech).

### Orbital Fibroblast Stimulation With TGF-β and Treatment

The orbital fibroblasts were stimulated with 10 ng/mL TGF-β1 for 24 hours^30^ and divided into 7 experimental groups: (1) TAO, (2) TAO + TGF-β1, (3) TAO + TGF-β1 + Tocilizumab (10 µg/mL, Toc), (4) TAO + TGF-β1 + Microspheres (MS, 262 µg/mL), (5) TAO + TGF-β1 + CD34Apt, (6) TAO + TGF-β1 + Tocilizumab-loaded Microspheres (Toc-MS, 297 µg/mL), and (7) TAO + TGF-β1 + CD34 Apt-modified Tocilizumab-loaded Microspheres (Toc-MS-CD34Apt, 297 µg/mL). The respective treatments were added to the culture medium for 48 hours simultaneously with TGF-β1. Various parameters were then measured to evaluate the effects of these treatments on the fibroblasts.

### Cell Counting Kit-8 Assay

A cell counting kit 8 (CCK-8) assay kit was used to examine cell viability based on metabolic activity.^31^^,^^32^ After different treatments, the orbital fibroblasts were further incubated with fresh medium supplemented with CCK-8 solution at 1:10 at 48 hours, followed by 2 hours of incubation. The OD value of each well at 450 nm was subsequently detected using a multi-function microplate reader (Bio-rad, Hercules, CA, USA).

### EdU Staining

EdU incorporation was used to assess cell proliferation.^33^^,^^34^ Cells were incubated with 50 µM EdU working solution (Ribobio, Guangzhou, China) for 2 hours. After discarding the culture medium, the cells were fixed with 1 mL of 4% paraformaldehyde for 15 minutes at RT. Following fixation, the cells were rinsed thrice with PBS, permeabilized with 0.5% Triton X-100 for 10 minutes, and then incubated with Apollo Reaction Buffer for 30 minutes at RT, in light-deprived conditions. The nuclei were stained with DAPI for 10 minutes, and the cells were washed thrice. The proliferating cells were observed as red fluorescent, and nuclei appeared blue under a fluorescence-inverted microscope.

### Scratch Wound Healing

Orbital fibroblasts were seeded into the six-well plate. A 200 µL pipette was used to create scratches after cells grew to 90% confluence. Fresh medium and various treatments were added to the wells after washing them with PBS. The images of scratches were captured at 0 hours and 48 hours after creating the scratches.^35^

### TAO Mice Modeling Procedure

Female BALB/c mice aged 6 to 8 weeks were used for this study, given their higher incidence of TAO, mirroring the condition's prevalence in female mice. The care and handling of all mice adhered strictly to the Guide for the Care and Use of Laboratory Animals, and all experimental procedures received approval from the Laboratory Animal Welfare and Ethics Committee of the Third Xiangya Hospital.

The mice were allocated into 5 groups (*n* = 6): control, TAO, TAO + anti-mmu-IL-6R, TAO + (anti-mmu-IL-6R)-MS, and TAO + (anti-mmu-IL-6R)-MS-CD34 Apt. TAO mice were induced using adenovirus expressing the TSHR A-subunit (Ad-TSHR289) injection, as previously described.^36^^–^^38^ All mice received intramuscular injections of about 10^9^ PFU of adenovirus within 50 µL of PBS, whereas the control group received a control blank adenovirus. The TAO mice model was established by administering 5 injections of Ad-TSHR289 at 3-week intervals. This extended protocol was designed to replicate the more pronounced orbital inflammation and tissue remodeling observed in TAO, including orbital fat expansion and fibrosis, which are hallmark signs of the disease.

After the 6-week immunization period (6 weeks after the last injection), the TAO mice received orbital injections of 24.57 mg/kg anti-mmu-IL-6R, 212 mg/kg anti-mmu-IL-6R-MS, and 212 mg/kg MR-16-1-MS-CD34 Apt every 2 weeks for 8 weeks. The mice were euthanized, and orbital tissues, blood samples, and thyroid tissues were then collected for analysis.

### Colocalization of CD34^+^ Orbital Fibroblast and Microspheres

Orbital fibroblasts from healthy controls and patients with TAO were incubated with either MS-Apt control-FAM or MS-CD34 Apt-FAM for 4 hours. After incubation, orbital fibroblasts underwent CD34 immunofluorescence staining, with secondary antibodies conjugated to a red fluorescent dye. Fluorescence microscopy was used to observe the colocalization of CD34^+^ orbital fibroblasts and MS-CD34 Apt-FAM. The CD34^+^ orbital fibroblasts were labeled with red fluorescence, Apts with green fluorescence, and cell nuclei with DAPI (blue). The colocalization and uptake process of MS-CD34 Apt-FAM into CD34^+^ orbital fibroblasts were recorded at 4 hours post-incubation using a confocal microscope.

### Enzyme-Linked Immunosorbent Test

The serum of mice was used for the enzyme-linked immunosorbent test (ELISA) to quantify the levels of thyroid-related hormones, including thyrotrophin receptor antibodies (TRAbs; ml001928, Shanghai Enzyme-linked Biotechnology Co., Ltd., Shanghai, China), thyroxine (T4; RXJ202844M, Ruixin, Quanzhou, China), and TSH (CSB-E05116m; Cusabio, Wuhan, China). All assays were conducted as directed by the manufacturer to ensure accurate quantification.

### Immunohistochemical Staining 

The orbital muscle and adipose tissues were subjected to paraffin embedding and cut into 5 µm sections. After dewaxing, the sections were subjected to antigen retrieval non-specific binding sites blocking with goat serum (GenePharm, China), endogenous peroxidase blocking with 0.3% H_2_O_2_, and then exposed to vimentin (10366-1-AP, Proteintech, Wuhan, China), α-SMA (14395-1-AP, Proteintech), or collagen I (14695-1-AP, Proteintech) antibody (dilution = 1:50–200). The sections were subsequently subjected to the polymer IgG detection system (ZSGB-BIO, Beijing, China) for a duration of 20 minutes at 37°C and thereafter stained with DAB solution and hematoxylin.

### Immunoblotting

Radioimmunoprecipitation assay buffer containing protease and phosphatase inhibitors (EpiZyme, Shanghai, China) was used to sample cells. A bicinchoninic acid protein assay kit (Beyotime) measured protein concentration. Equal amounts of total protein were electro-transferred onto polyvinylidene fluoride membranes (Millipore, Darmstadt, Germany) from sodium dodecyl sulfate–polyacrylamide gels (8% or 12%). Membranes were incubated in QuickBlock Blocking Buffer (Genscript, Nanjing, China) for 15 minutes to prevent nonspecific bindings. Primary antibodies against p-STAT3 (CSB-RA022812A727phHU, Cusabio), STAT3 (10253-2-AP, Proteintech), FGF2 (11234-1-AP, Proteintech), FGFR2 (CSB-PA000992, Cusabio), and PCNA (10205-2-AP, Proteintech) were then incubated overnight. The endogenous control was GAPDH. The membranes were treated with horseradish peroxidase-conjugated anti-mouse or anti-rabbit secondary antibodies for 2 hours after 3 times of TBST washes. The membrane was finally examined with a Millipore chemiluminescence Western blot kit and an automatic imaging system (Tanon Science & Technology Co., Ltd., Shanghai, China). ImageJ software (NIH, Bethesda, MD, USA) evaluated the protein band density.

### Statistical Analysis

The cell experiments were performed for three biological replicates. The animal experiments were performed for six biological replicates. The statistical analysis and data processing were conducted using GraphPad Prism version 9.5.0 (GraphPad Software, Inc., La Jolla, CA, USA). The data are reported in the form of the mean value plus or minus the standard deviation (SD). A student's *t-*test was used to assess continuous data that followed a normal distribution in two groups, whereas the Mann-Whitney test was used for continuous data that did not adhere to a normal distribution. When comparing multiple groups, the 1-way ANOVA followed by the Tukey test or Dunnett T3 test was used. A *P* value below 0.05 was considered statistically significant.

## Results

### Selection and Verification of CD34 Apt Affinity for Human Orbital Fibroblasts

**Figure 1.**
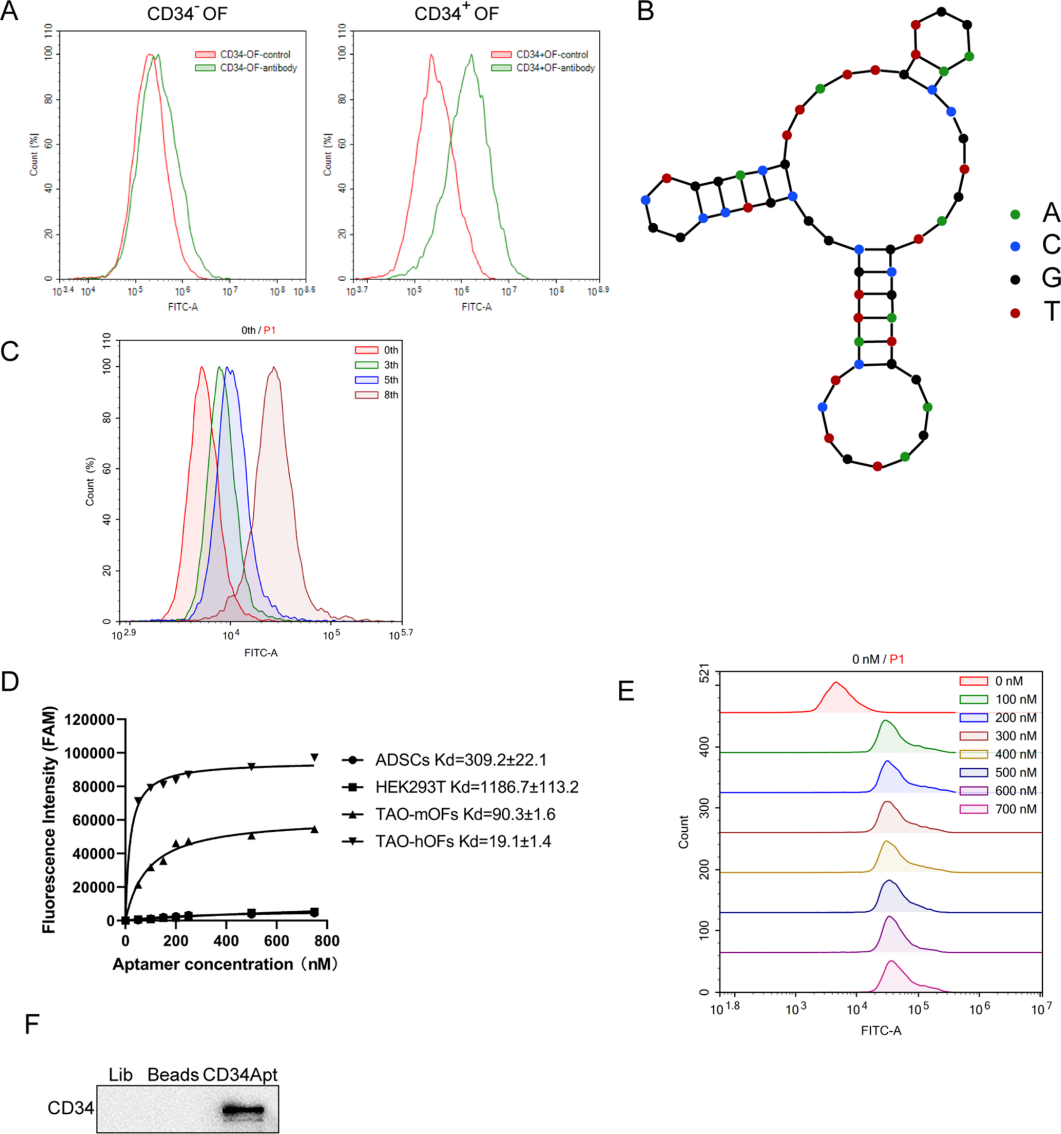
**Verification of CD34** **Aptamer (CD34** **Apt) affinity for human orbital fibroblasts.** (**A**) Flow cytometric sorting of human CD34^−^ and CD34^+^ orbital fibroblasts. Representative flow cytometry plots showing the sorting of human orbital fibroblasts into CD34^+^ and CD34^−^ populations. (**B**) Predicted secondary structure of the CD34 Aptamer. The secondary structure of the CD34 Aptamer, as predicted using bioinformatics tools. (**C**) Enrichment of CD34 Aptamer binding after 8 rounds of SELEX screening. Flow cytometry analysis demonstrating the fluorescence intensity of the CD34 Aptamer after eight rounds of selection, indicating successful enrichment. (**D,** **E**) Affinity verification of CD34 Aptamer for various cell types. Flow cytometry analysis showing the binding affinity of the FAM-labeled CD34 Aptamer to primary mouse orbital fibroblasts, primary human orbital fibroblasts, HEK293 cells, and human adipose-derived mesenchymal stem cells (CD34^−^). The mean fluorescence intensity (MFI) is plotted against the aptamer concentration, and the data are modeled using the equation *Y* = *B*_max _*X*/(*K_d_* + *X*). The flow cytometer histogram of TAO hOFs was shown in (**E**). (**F**) Aptamer-mediated pull-down assay. Pull-down assay results verify the interaction between the CD34 Aptamer and CD34 protein, confirming the specific binding of the aptamer to the target protein (*N* = 3 biological replicates).

Before aptamer screening, CD34^+^ and CD34^−^ orbital fibroblast were sorted using flow cytometric sorting for positive/negative selection of CD34 Apt. Representative flow cytometry plots of human orbital fibroblasts into CD34^−^ and CD34^+^ populations were shown in Figure 1A. The CD34Apt was designed as described, and its secondary structure is predicted and shown in Figure 1B. The binding affinity of CD34 Apt to CD34^+^ orbital fibroblast was evaluated; flow cytometry analysis demonstrated that a peak in fluorescence intensity after 8 rounds of selection, indicating successful enrichment (Fig. 1C). The binding assays revealed that CD34 Apt exhibited high specificity and affinity to both human and mouse orbital fibroblasts, with a Kd of 19.1 ± 1.4 nM for human and 90.3 ± 1.6 nM for mouse orbital fibroblasts (Fig. 1D). Further validation of CD34 Apt binding to CD34^+^ orbital fibroblasts was performed through flow cytometry, confirming targeted binding (Fig. 1E). Apt-mediated pull-down assay verified the interaction between the CD34 Apt and CD34 protein, confirming the specific binding of the aptamer to the target protein in orbital fibroblasts (Fig. 1F).

### Preparation and Characterization of CD34 Aptamer-Coupled Tocilizumab Microspheres

**Figure 2.**
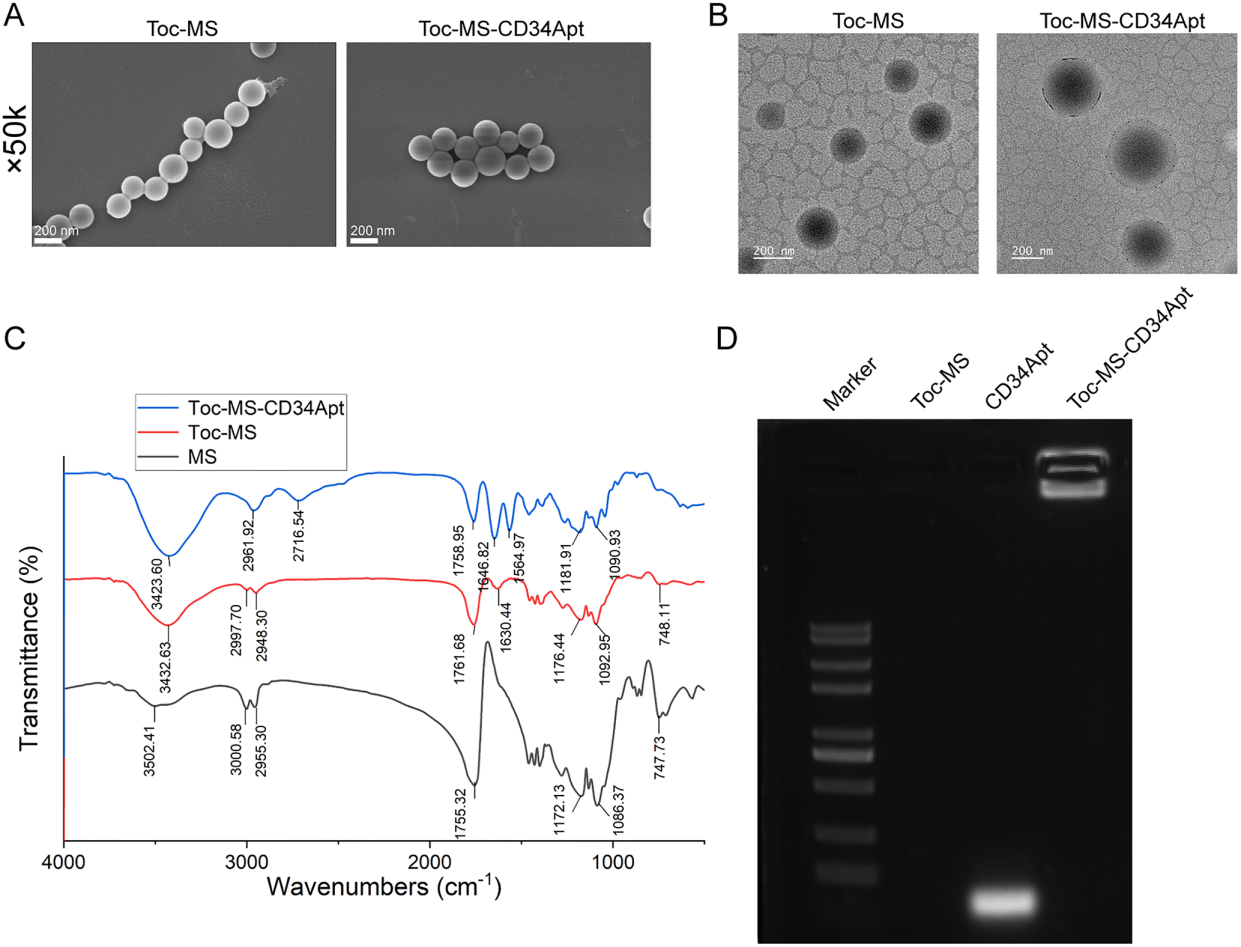
**Preparation and characterization of CD34 Aptamer-coupled Tocilizumab microspheres** **.** (**A**) Scanning electron microscopy (SEM) images showing the surface structure and size uniformity of the microspheres of Tocilizumab (Toc)-loaded microspheres (MS; Toc-MS) and CD34 Aptamer-modified Tocilizumab-loaded microspheres (Toc-MS-CD34 Apt). (**B**) Transmission electron microscopy (TEM) images showing the internal structure of MS. (**C**) Fourier transform infrared spectroscopy (FTIR) analysis was performed to evaluate the surface modification of microspheres to confirm the presence of Toc and CD34 Aptamers. (**D**) Agarose gel electrophoresis was performed to access the distribution of CD34 Aptamers on the surface of the microspheres, comparing the migration patterns of CD34Apt alone and CD34Apt-modified Toc-MS.

PLGA-based microspheres coupled with CD34 Apt were subsequently prepared and characterized. scanning electron microscopy (SEM) and TEM images revealed that both Toc-MS and Toc-MS-CD34 Apt microspheres were uniform in size (approximately 0.3 µm in diameter) and maintained smooth surfaces (Figs. 2A, 2B). FTIR spectra indicated successful modification of the microspheres: MS showed a characteristic peak of the carboxylic acid of PLGA at 1755.32 cm^−1^; Toc-MS showed an Amide I peak at 1630 cm^−1^, confirming Toc loading; whereas Toc-MS-CD34 Apt exhibited additional peaks at 1646 cm^−1^ (Amide I) and 1564.97 cm^−1^ (imine), indicating CD34Apt conjugation (Fig. 2C). The electrophoresis results demonstrated distinct bands for the CD34 Apts. However, after conjugation to the microspheres, the modified Apts on Toc-MS-CD34 Apt showed reduced mobility due to the increased molecular weight, thereby confirming the successful attachment of CD34 Apts to the microsphere surface (Fig. 2D).

### Drug Release and Degradation Rates of Toc-MS and Toc-MS-CD34 Apt In Vitro

**Figure 3.**
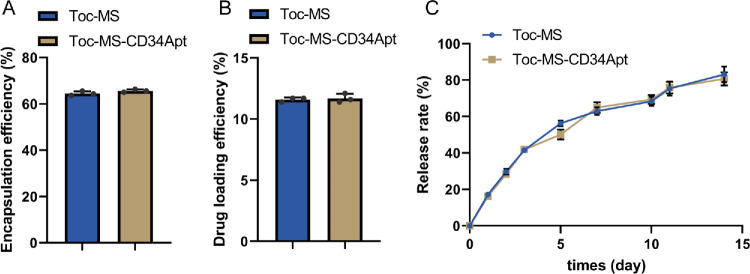
**Drug release and degradation rates of Tocilizumab-loaded microspheres (Toc-MS) and CD34** **Aptamer-modified Tocilizumab-loaded microspheres (Toc-MS-CD34** **Apt) in vitro.** (**A**) The encapsulation efficiency (EE) was calculated using the formula *EE* = (*W_e_*/*W*_0_) × 100%, where *W_e_* is the mass of the drug encapsulated in the microspheres and *W*_0_ is the theoretical mass of the drug. (**B**) The drug loading efficiency (DL) of the microspheres was calculated using the formula *W*_e_, where *W*_e_ is the mass of the drug encapsulated in the microspheres and *W_m_* is the total mass of the drug-loaded microspheres. (**C**) The release of Tocilizumab from the microspheres was measured using an ELISA kit (*N* = 3 biological replicates).

The DL efficiency and EE were calculated for Toc-MS and Toc-MS-CD34 Apt. The DL for Toc-MS was 11.60%, and for Toc-MS-CD34 Apt it was 11.70%, whereas EE was 64.46% for Toc-MS and 65.62% for Toc-MS-CD34 Apt (Figs. 3A, 3B). The in vitro release profile showed a sustained release of Toc from both MS types, with a release of 83.06% for Toc-MS and 80.81% for Toc-MS-CD34 Apt by day 15 (Fig. 3C).

### Isolation and Identification of Non-TAO and TAO Orbital Fibroblasts

**Figure 4.**
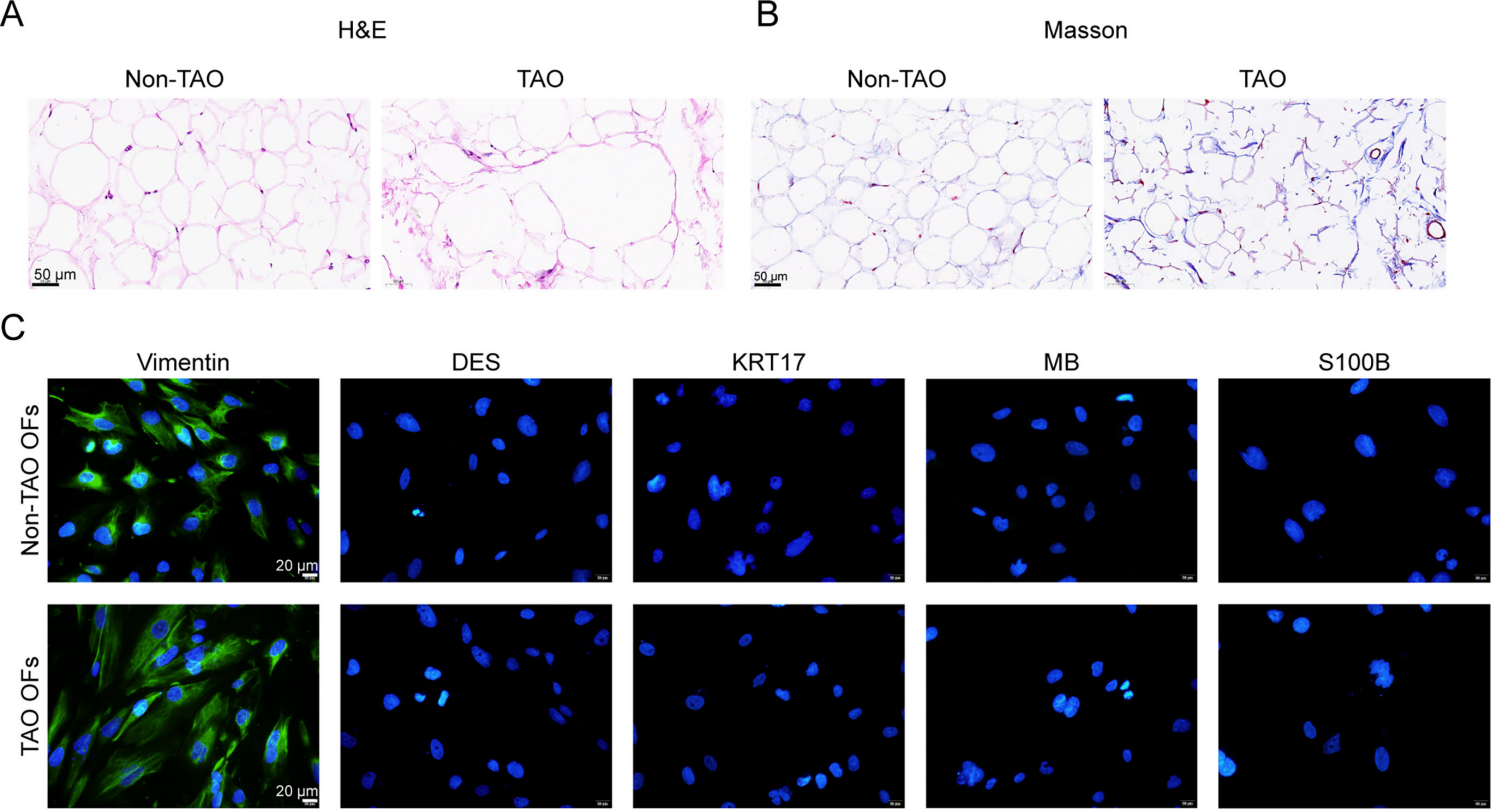
**Isolation and identification of non-TAO and TAO orbital fibroblasts.** (**A, B**) Orbital samples were collected from non-TAO donors and patients with TAO and verified for histopathological features using H&E and Masson staining. (**C**) Orbital fibroblasts were isolated from non-TAO and TAO orbital samples and verified using immunofluorescent staining (IF staining) to detect the levels of vimentin, desmin (DES), keratin 17 (KRT17), myoglobin (MB), and S100B.

For the isolation of orbital fibroblasts, clinical orbital tissue samples were harvested from non-TAO donors or patients with TAO. H&E staining of orbital tissues revealed widened interstitial spaces in orbital fat and significant degeneration of extraocular muscle fibers with vacuole formation in TAO tissues compared with healthy controls (Fig. 4A). Masson's trichrome staining showed increased collagen deposition and fibrosis in the intermuscular connective tissue of patients with TAO (Fig. 4B). The primary orbital fibroblasts were then successfully isolated and cultured from the orbital tissues of both patients with TAO and non-TAO donors. To confirm the identity of primary orbital fibroblasts, immunofluorescent (IF) staining was performed for several markers, including vimentin, DES, KRT17, MB, and S100B (Fig. 4C). IF staining results indicated that vimentin was strongly positive in both non-TAO and TAO orbital fibroblasts, confirming the fibroblastic nature of the cells. In contrast, the cells stained negatively for DES, KRT17, MB, and S100B (see Fig. 4C).

### CD34 Apt-Coupled Tocilizumab Microspheres Inhibit CD34^+^ Orbital Fibroblast Activation In Vitro

**Figure 5.**
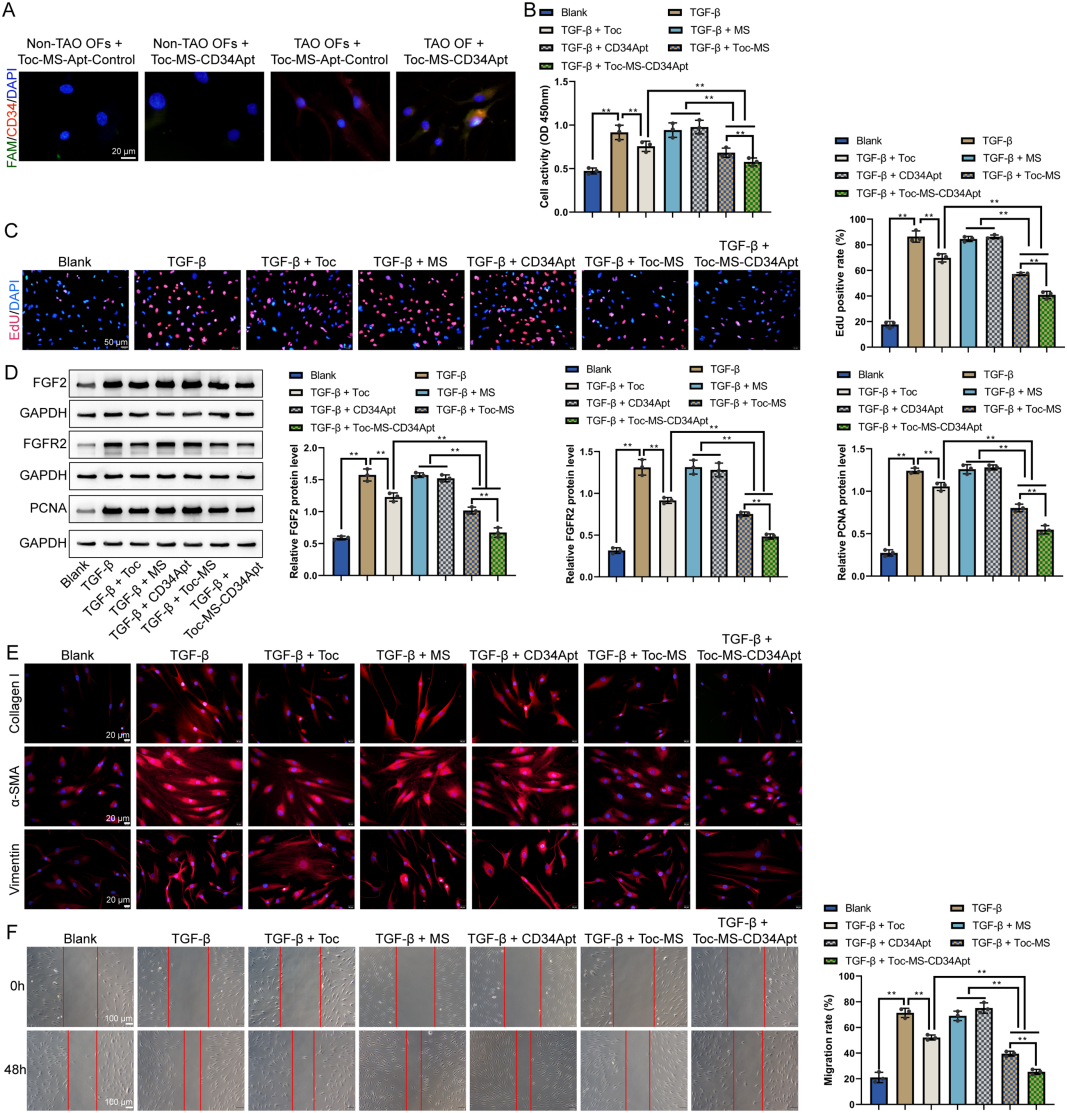
**CD34 Apt-coupled Tocilizumab microspheres inhibit CD34^+^ orbital fibroblast activation in vitro.** (**A**) Colocalization of CD34^+^ orbital fibroblast and microspheres was determined by IF staining. Orbital fibroblasts from normal and TAO orbital tissues were incubated with either MS-Apt control-FAM or MS-CD34 Apt-FAM for 4 hours. CD34 IF staining (*red*) was performed on orbital fibroblasts. Fluorescence microscopy was used to observe the colocalization of CD34^+^ orbital fibroblasts and MS-CD34 Apt-FAM (*green*), with DAPI staining the nuclei (*blue*). Then, TAO orbital fibroblasts were stimulated with TGF-β1 for 24 hours and treated with different formulations accordingly for 48 hours: (1) TAO + Blank, (2) TAO + TGF-β1, (3) TAO + TGF-β1 + Toc (Tocilizumab), (4) TAO + TGF-β1 + MS (empty microsphere), (5) TAO + TGF-β1 + CD34 Apt, (6) TAO + TGF-β1 + Toc-MS, and (7) TAO + TGF-β1 + Toc-MS-CD34 Apt. (**B, C**) Fibroblasts’ viability and proliferation were measured using the CCK-8 assay **B** and EdU staining **C**. (**D**) The levels of fibroblast activation factors, including FGF2, FGFR2, and cell proliferation marker and PCNA were detected by immunoblotting. (**E**) IF staining was performed to detect the expression of extracellular matrix proteins α-SMA, collagen I, and vimentin in the treated fibroblasts. (**F**) The migration of treated fibroblasts was assessed using a wound-healing assay (*N* = 3 biological replicates). **P < 0.01.

Next, to evaluate the specific effects of CD34 Apt-coupled Toc MS on CD34^+^ orbital fibroblasts, non-TAO and TAO orbital fibroblasts were co-cultured with control aptamer or CD34 Apt-modified microspheres. IF staining revealed that in the control groups (Normal + MS-Apt control and Normal + MS-CD34 Apt), no CD34^+^ orbital fibroblasts were present, and FAM fluorescence was minimal. In the TAO + MS-Apt control group, a limited distribution of FAM fluorescence was observed within the cells. However, in the TAO + MS-CD34 Apt group, the MS-CD34 Apt-FAM fluorescence was specifically colocalized with CD34^+^ orbital fibroblasts, indicating targeted binding (Fig. 5A). TAO orbital fibroblasts were subsequently treated with TGF-β1 and various treatments: Toc, Toc-MS, and Toc-MS-CD34 Apt. The CCK-8 assay (Fig. 5B) and EdU staining (Fig. 5C) demonstrated that TGF-β1 significantly stimulated the cell viability and cell proliferation of TAO orbital fibroblasts. The addition of MS and CD34 Apt caused no significant changes in cell viability and cell proliferation. In contrast, Toc, Toc-MS, and Toc-MS-CD34 Apt showed varying degrees of inhibition on cell viability and cell proliferation when compared with the TAO orbital fibroblasts + TGF-β1 group, with Toc-MS-CD34 Apt exhibiting the most pronounced inhibitory effect (see Figs. 5B, 5C). Western blot analysis showed that TGF-β1 stimulation upregulated the expression of key fibroblast activation factors, including FGF2, FGFR2, and cell proliferation markers Ki67. Treatment with Toc, Toc-MS, and Toc-MS-CD34 Apt significantly reduced the expression of these markers, with Toc-MS-CD34 Apt exhibiting the most significant reduction (Fig. 5D). Moreover, IF staining of ECM proteins α-SMA, collagen I, and vimentin revealed that TGF-β1 increased their expression in TAO fibroblasts. In contrast, Toc, Toc-MS, and Toc-MS-CD34 Apt reduced their levels, with Toc-MS-CD34 Apt showing the greatest reduction effect (Fig. 5E). The wound healing assay confirmed that TGF-β1 enhanced fibroblast migration. Still, Toc-MS and Toc-MS-CD34 Apt notably inhibited migration, with Toc-MS-CD34 Apt showing the most significant reduction (Fig. 5F).

### Preparation and Characterization of CD34 Aptamer-Coupled Anti-mmu-IL-6R Microspheres

**Figure 6.**
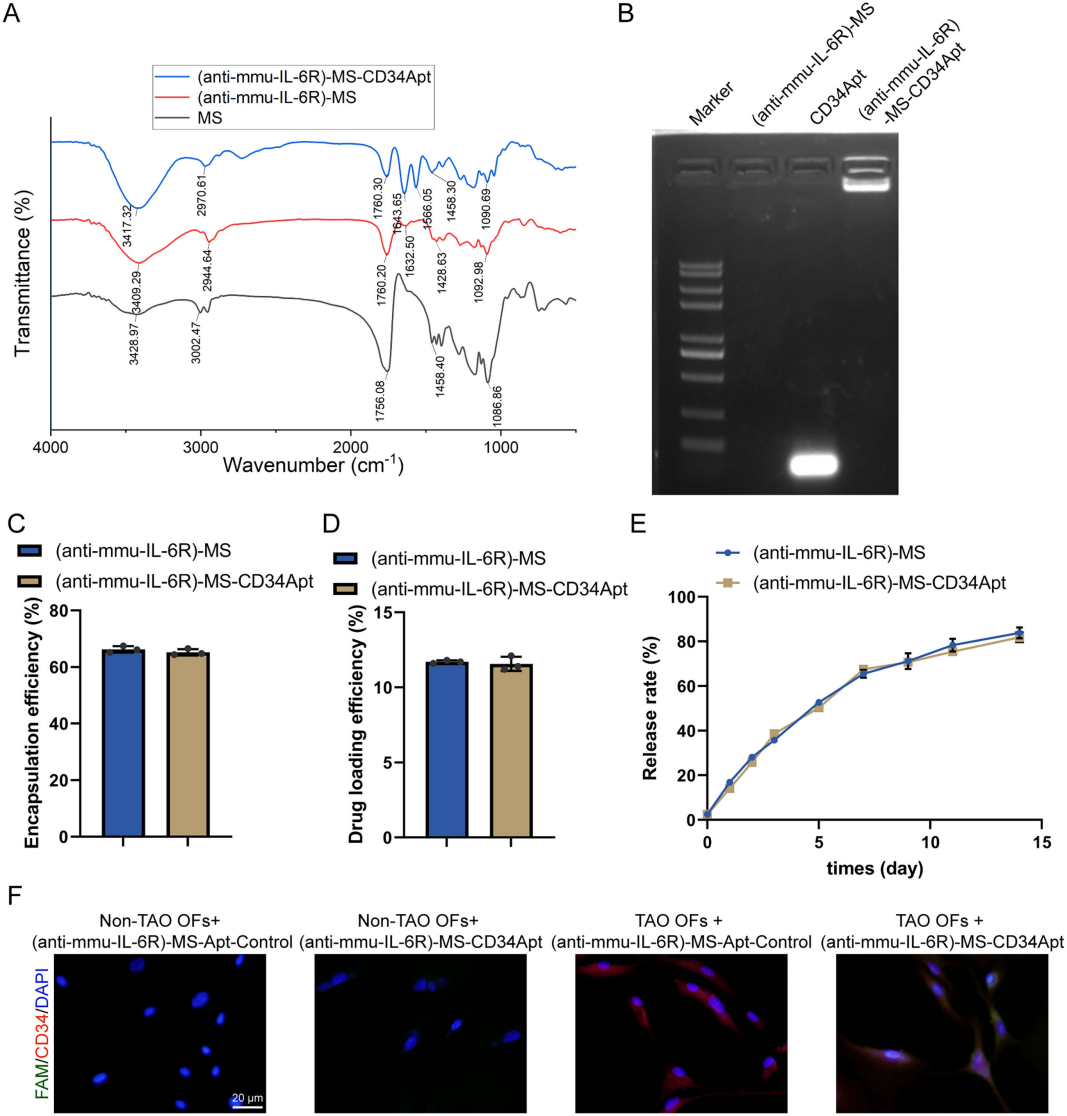
**Characterization of (anti-mmu-IL-6R)-MS and CD34 aptamer-modified (anti-mmu-IL-6R)-MS-CD34** **Apt.** (**A**) FTIR analysis was performed to evaluate the surface modification of microspheres to confirm the presence of CD34 aptamers. (**B**) Agarose gel electrophoresis was performed to access the distribution of CD34 aptamers on the surface of the microspheres, comparing the migration patterns of CD34 Apt alone and CD34 Apt-modified (anti-mmu-IL-6R)-MS. (**C**) The encapsulation efficiency (EE) was calculated using the formula *EE* = (*W_e_*/*W*_0_) × 100%, where *W_e_* is the mass of the drug encapsulated in the microspheres and *W*_0_ is the theoretical mass of the drug. (**D**) The drug loading efficiency (DL) of the microspheres was calculated using the formula *DL* = (*W_e_*/*W_m_*) × 100%, where *W_e_* is the mass of the drug encapsulated in the microspheres and *W*_m_ is the total mass of the drug-loaded microspheres. (**E**) The release of anti-mmu-IL-6R from the microspheres was measured using an ELISA kit. (**F**) Mouse orbital fibroblasts from normal and TAO orbital tissues were incubated with either MS-Apt control-FAM or MS-CD34 Apt-FAM for 4 hours. CD34 IF staining (*red*) was performed on orbital fibroblasts. Fluorescence microscopy was used to observe the colocalization of CD34^+^ orbital fibroblasts and MS-CD34 Apt-FAM (*green*), with DAPI staining the nuclei (*blue*; *N* = 3 biological replicates).

First, the characters of (anti-mmu-IL-6R)-MS and (anti-mmu-IL-6R)-MS-CD34 Apt were determined by FTIR and Agarose gel electrophoresis (Figs. 6A, 6B). FTIR analysis was performed to evaluate the surface modification of microspheres to confirm the presence of CD34 Apts (see Fig. 6A). Agarose gel electrophoresis was performed to assess the distribution of CD34 Apts on the surface of the microspheres (see Fig. 6B). The drug EE and DL were also evaluated and calculated. Figure 6C shows that the EE for (anti-mmu-IL-6R)-MS was 66.23% and 65.20% for (anti-mmu-IL-6R)-MS-CD34 Apt. Figure 6D shows that the drug loading efficiency was 11.7% for (anti-mmu-IL-6R)-MS and 11.6% for (anti-mmu-IL-6R)-MS-CD34 Apt. The release rate reached 83.90% for (anti-mmu-IL-6R)-MS and 81.86% for (anti-mmu-IL-6R)-MS-CD34 Apt at day 15 (Fig. 6E). Furthermore, (anti-mmu-IL-6R)-MS-CD34 Apt specifically bound to TAO orbital fibroblasts, as demonstrated by the binding assays (Fig. 6F).

### TAO Mouse Model Establishment and Validation

**Figure 7.**
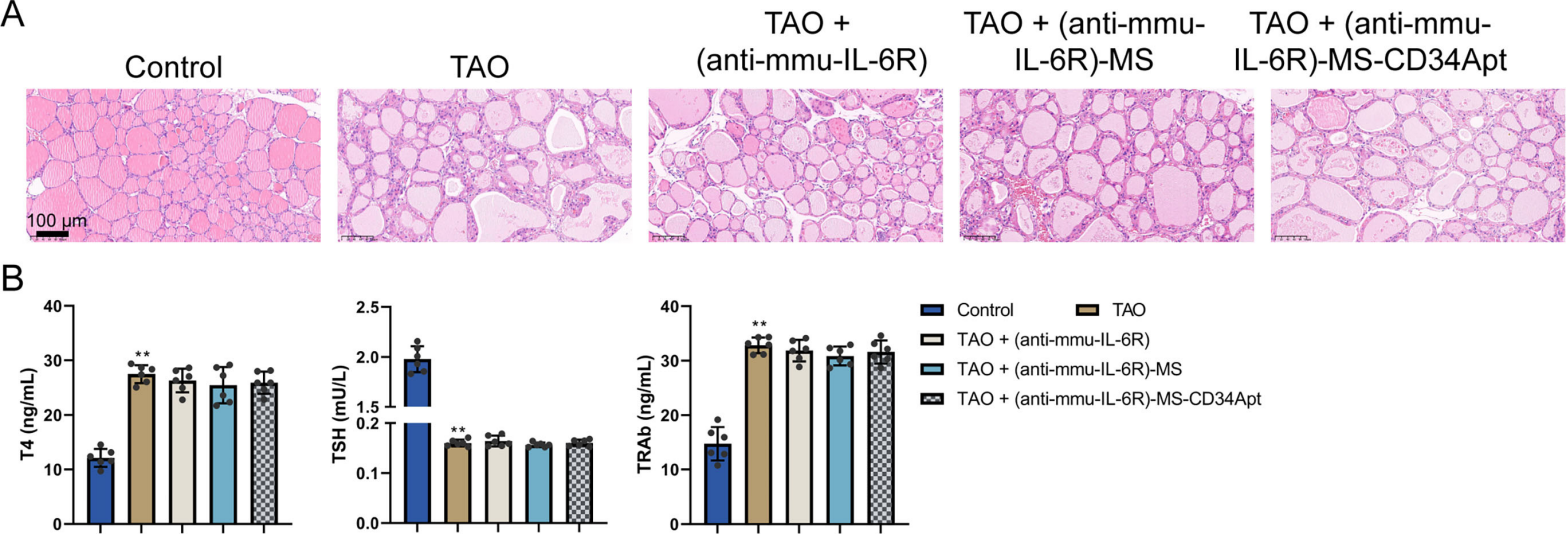
**The TAO mouse model establishment and validation** **.** Model mice were divided into five groups: Control, TAO, TAO + (anti-mmu-IL-6R), TAO + (anti-mmu-IL-6R)-MS, and TAO + (anti-mmu-IL-6R)-MS-CD34 Apt; mice in each group received corresponding treatment. (**A**) The histopathological alterations in thyroid tissues of TAO mice were detected by H&E staining. (**B**) Mouse serum T4, TSH, and TRAb levels in TAO mice were determined using ELISA (*N* = 6 biological replicates). **P < 0.01 compared to the healthy control group.

The TAO model was established in mice as described and subsequently validated. H&E-stained thyroid tissue sections exhibited characteristic changes consistent with hyperthyroidism, including follicular cell hyperplasia and colloid depletion, confirming the presence of hyperthyroidism in the TAO mice (Fig. 7A). The results of serum ELISA indicated significantly elevated levels of T4 and TRAb, whereas the TSH level was markedly restrained in the TAO mice compared to the healthy controls, thereby further validating the hyperthyroid status of the TAO mice (Fig. 7B).

### Therapeutic Effects of CD34 Apt-Coupled Mouse Anti-IL-6R Microspheres in TAO Mice

**Figure 8.**
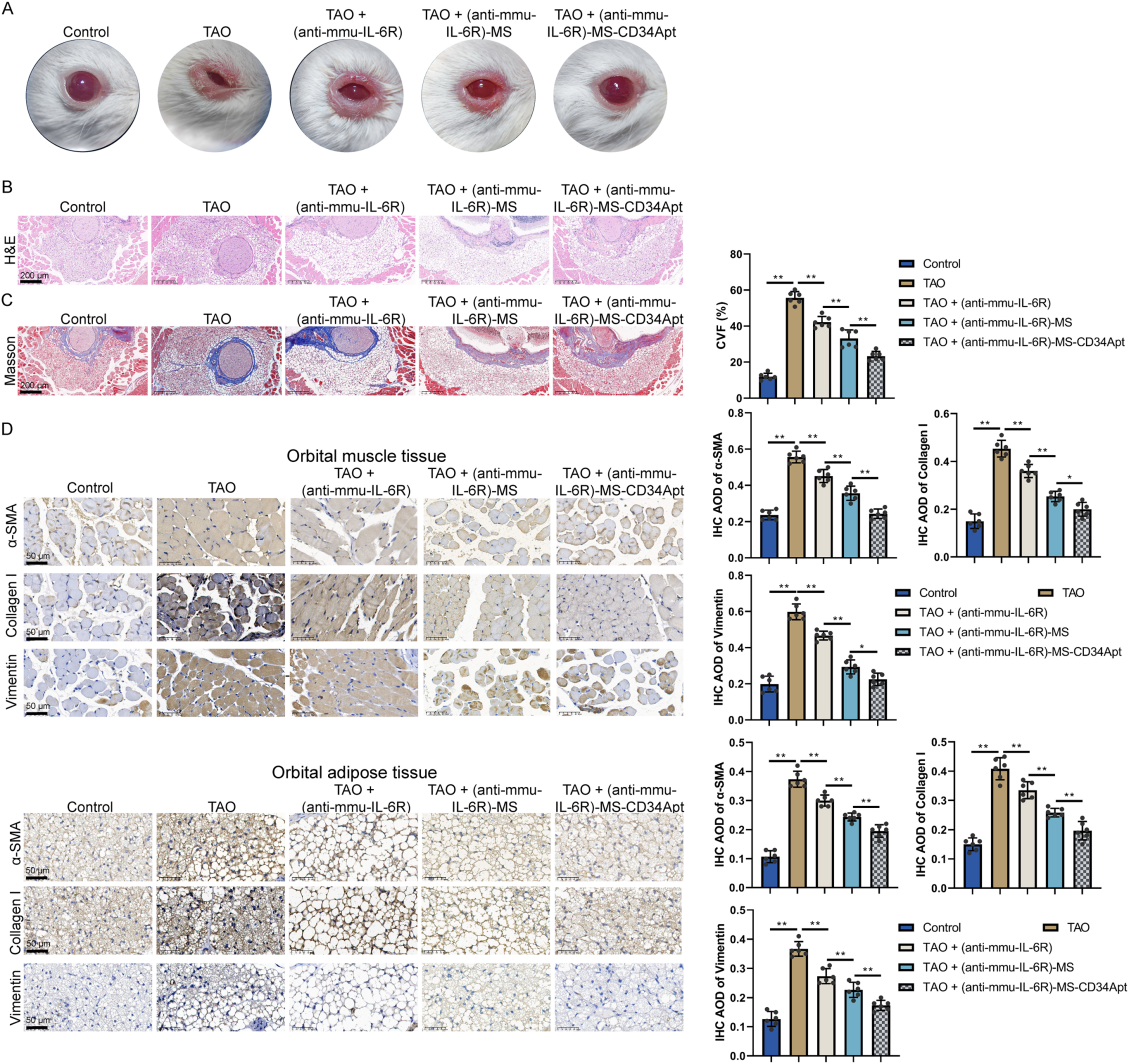
**Therapeutic effects of CD34 Apt-coupled mouse anti-IL-6R microspheres in TAO mice** **.** Model mice were divided into five groups: Control, TAO, TAO + (anti-mmu-IL-6R), TAO + (anti-mmu-IL-6R)-MS, and TAO + (anti-mmu-IL-6R)-MS-CD34 Apt; mice in each group received corresponding treatment. (**A**) Photographs of the left eyeballs of model mice. (**B**) H&E staining of mouse orbital muscle and adipose tissues. (**C**) Masson's trichrome staining of mouse orbital muscle and adipose tissues to assess fibrosis. (**D**) Immunohistochemical (IHC) staining for ECM proteins (α-SMA, collagen I, and vimentin) in mouse orbital muscle and adipose tissues (*N* = 6 biological replicates). *P < 0.05, **P < 0.01.

First, the effects of CD34 Apt-coupled anti-mmu-IL-6R microspheres on thyroid function in TAO mice were investigated (see Figs. 7A, 7B). No significant changes were observed in the pathological thyroid tissue alterations (see Fig. 7A) or in serum levels of T4, TRAb, and TSH (see Fig. 7B) following treatment with (anti-mmu-IL-6R), (anti-mmu-IL-6R)-MS, or (anti-mmu-IL-6R)-MS-CD34 Apt. Subsequently, the ocular effects of CD34 Apt-coupled anti-mmu-IL-6R microspheres in TAO mice were evaluated. The left eyeballs of the mice in the different experimental groups saw visible changes in the treated groups compared to the healthy controls, with the (anti-mmu-IL-6R), (anti-mmu-IL-6R)-MS, and (anti-mmu-IL-6R)-MS-CD34 Apt groups showing a reduction in ocular inflammation compared to TAO mice (Fig. 8A). H&E staining of orbital muscle and adipose tissues revealed prominent tissue remodeling and inflammation in the TAO group, which were significantly reduced in the treatment groups. The most notable improvement was observed in the (anti-mmu-IL-6R)-MS-CD34 Apt group, which exhibited minimal inflammation and tissue damage (Fig. 8B). Masson's trichrome staining further indicated reduced fibrosis in treated groups, with the (anti-mmu-IL-6R)-MS-CD34 Apt group showing the greatest reduction in collagen deposition, suggesting a strong therapeutic effect (Fig. 8C). Immunohistochemical (IHC) staining for ECM proteins α-SMA, collagen I, and vimentin showed elevated expression in the TAO group, correlating with increased fibrosis. Treatment with (anti-mmu-IL-6R), (anti-mmu-IL-6R)-MS, and (anti-mmu-IL-6R)-MS-CD34 Apt led to a significant decrease in ECM protein levels, with the most significant reduction observed in the (anti-mmu-IL-6R)-MS-CD34 Apt group (Fig. 8D).

### Tocilizumab/(anti-mmu-IL-6R) Inhibits STAT3 Pathway Activation in Fibroblasts and Orbital Tissues

**Figure 9.**
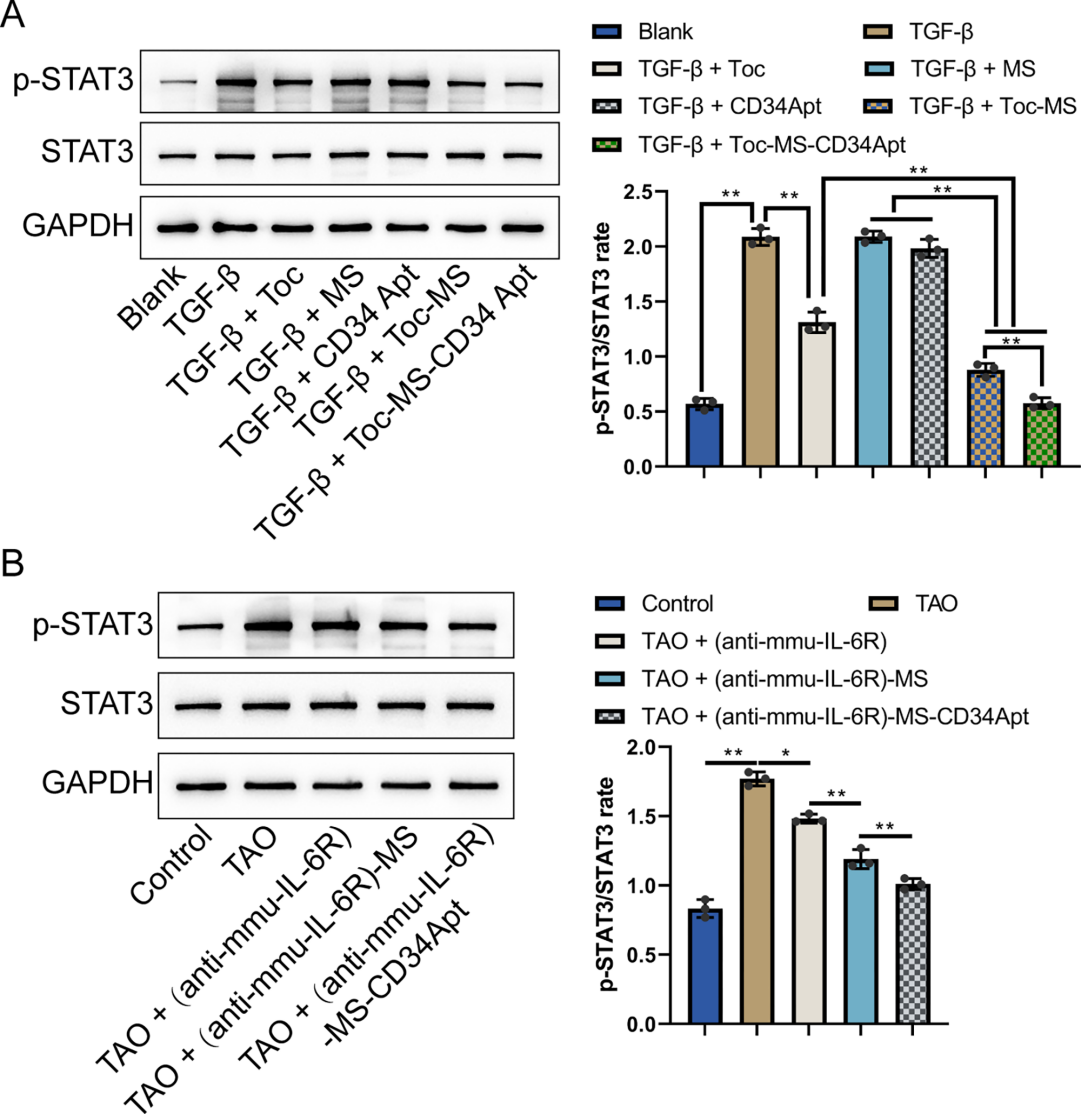
**Tocilizumab/(anti-mmu-IL-6R) inhibits STAT3 pathway activation in fibroblasts and orbital tissues** **.** (**A**) The protein levels of p-STAT3 and STAT3 levels in differentially treated human orbital fibroblasts were determined using immunoblotting. (**B**) The protein levels of STAT3 and p-STAT3 levels in model mice orbital tissues were determined using immunoblotting (*N* = 3 biological replicates). *P < 0.05, **P < 0.01.

To assess the impact of Toc/(anti-mmu-IL-6R) on STAT3 signaling, immunoblotting was performed to measure levels of phosphorylated STAT3 (p-STAT3) and total STAT3 in TAO human orbital fibroblasts and orbital tissues from the TAO mouse model. In human TAO orbital fibroblasts, the levels of p-STAT3 were significantly elevated after TGF-β·stimulation compared to controls. Incubation with Toc, Toc MS, and Toc MS-CD34 Apt resulted in a marked decrease of p-STAT3 levels, with the most substantial inhibition observed in the Toc MS-CD34 Apt group treated TAO orbital fibroblasts (Fig. 9A). Similarly, in mouse orbital tissues, high p-STAT3 levels were observed in the untreated TAO group, indicating STAT3 pathway activation. This activation was significantly reduced in the treatment groups, particularly in the (anti-mmu-IL-6R)-MS-CD34 Apt group, demonstrating the effectiveness of the treatment in inhibiting STAT3 phosphorylation (Fig. 9B).

## Discussion

Our study focused on screening Apts designed to selectively target CD34^+^ orbital fibroblasts, given their crucial role in TAO pathogenesis.^12^^,^^39^ Beyond their established role, the prevalence and specific characteristics of CD34^+^ orbital fibroblasts across different patient populations and disease stages further underscore their importance as a therapeutic target. The number of circulating CD34^+^ fibrocytes is significantly elevated in patients with active TAO, particularly in GD.^10^^,^^11^ These cells infiltrate the orbit and differentiate into CD34^+^ orbital fibroblasts, a cell type notably absent in healthy tissues, highlighting their specific role in TAO pathology. Studies have shown increased CD34 expression in both severe and mild Graves’ orbitopathy (GO), with a significant correlation between CD34 expression and other inflammatory and fibrotic markers.^40^ Human fibrocytes, including CD34^+^ fibroblasts, have also been shown to co-express thyroglobulin and thyrotropin receptors. This subset of CD34^+^ orbital fibroblasts exhibits enhanced pro-inflammatory activity and high TSHR expression, producing cytokines, such as IL-6 and TNF-α, in response to TSH and TSIs. Specifically, thyrotropin has been shown to regulate IL-6 expression in CD34^+^ fibrocytes, clearly delineating its cAMP-independent actions.^11^^,^^12^ Thyrotropin makes them a critical and highly relevant target for therapeutic intervention, as directly targeting these cells with CD34-specific agents can enhance treatment efficacy by reducing inflammation and fibrosis. The consistent finding of CD34 expression as a marker of orbital tissue remodeling further supports their role as a promising therapeutic target.

Through successive rounds of selection, a significant increase in the affinity of the CD34 Apt (CD34 Apt) for CD34^+^ orbital fibroblasts was observed, as demonstrated by elevated fluorescence levels. The binding affinity of CD34 Apt was quantified using equilibrium dissociation constants (Kd), with lower Kd values signifying stronger interactions, which are essential for the effectiveness of Apts in targeted therapies.^41^^,^^42^ The precise specificity of CD34 Apt was validated using Apt-mediated pull-down experiments, which confirmed its selective binding to CD34^+^ orbital fibroblasts. Crucially, the screening and validation of CD34 Apt have paved the way for the development of targeted therapies using CD34 Apt-modified MS. When coupled with the Apt, the MS exhibited significantly enhanced binding to CD34^+^ fibroblasts compared to unmodified microspheres, demonstrating superior targeting capability. This targeted approach is particularly valuable in the context of TAO, where CD34^+^ fibroblasts play a central role in the pathological processes of fibrosis and inflammation in TAO.^8^^,^^9^ In addition, for in vivo studies, microspheres loaded with mouse-derived IL-6R monoclonal antibody (anti-mmu-IL-6R) were developed to circumvent the issue of Toc not binding to mouse IL-6R.^43^

Due to their unique 3D folding based on their sequence, Apts can bind target molecules with high affinity and specificity, often surpassing that of antibodies.^44^^,^^45^ In our study, the following in vitro functional analysis further confirmed the binding affinity and efficacy of CD34 Apt-coupled Tocilizumab microspheres in mitigating TGF-β-induced changes in CD34^+^ orbital fibroblasts. The binding efficiency to CD34^+^ orbital fibroblasts was markedly higher for CD34 Apt-modified MS to target TAO orbital fibroblasts, underscoring their enhanced targeting capabilities. Importantly, these Apt-modified microspheres influenced key markers of fibroblast activation and fibrotic changes, including vimentin, α-SMA, and collagen I. Vimentin and α-SMA are cytoskeletal proteins that increase during fibroblast activation, signifying a transition toward a myofibroblast phenotype, a hallmark sign of fibrosis.^46^^,^^47^ Similarly, collagen I is an extracellular matrix component whose upregulation indicates heightened fibrotic activity, critical in the tissue remodeling seen in TAO.^48^ Further analysis demonstrated that the CD34 Apt-modified microspheres effectively suppressed the proliferation of TAO orbital fibroblasts, another characteristic of activated fibroblasts.^49^ These findings were consistent with the observed effects on fibroblast migration, where CD34 Apt-modified MS significantly suppressed the migration of TGF-β1-stimulated TAO fibroblasts. This finding suggests a strong potential for these modified MS to inhibit fibroblast mobility and fibrosis, thereby mitigating the tissue remodeling associated with TAO.

In evaluating the in vivo efficacy of CD34 Apt-coupled anti-IL-6R microspheres, a TAO model was established, and CD34 Apt-modified anti-IL-6R MS showed significant therapeutic effects on TAO mice. The TAO mice treated with CD34 Apt-modified microspheres exhibited notable improvements in tissue remodeling, inflammation, and ECM deposition. Specifically, Masson's trichrome staining revealed reduced collagen deposition in orbital muscle and adipose tissues, and the reduction in ECM markers further confirmed the microspheres’ ability to reduce fibrotic activity. The observed reduction in collagen deposition and ECM markers, consistent with established indicators of reduced fibrotic activity in TAO,^50^ suggests that our Apt-guided system is effective in mitigating tissue remodeling. The improvements were most pronounced in the group treated with CD34 Apt-modified MS, highlighting the efficacy of this targeted approach in reducing fibrosis and inflammation in vivo. Regarding the underlying mechanism, the impact of CD34 Apt-coupled and non-modified (anti-mmu-IL-6R)-MS on the STAT3 signaling pathway was investigated. The STAT3 signaling is known to play a critical role in inflammation and fibrosis.^51^ Previously, STAT3 has been reported to affect proinflammatory cytokine production, oxidative stress responses, and adipogenesis in an in vitro model of GO, suggesting that STAT3 mediates GO pathology, and that modulating STAT3 expression may have therapeutic potential against GO.^52^ In this study, elevated p-STAT3 levels in the untreated TAO fibroblasts and orbital tissues indicated active STAT3 signaling. The treatments with the CD34 Apt-modified microspheres significantly reduced p-STAT3 levels, suggesting that the targeted therapy effectively inhibits the STAT3 pathway. This finding was consistent across both human orbital fibroblasts and the TAO mouse model, reinforcing the potential of CD34 Apt-modified MS to modulate key inflammatory and fibrotic pathways.

The strength of the model lies in its recapitulation of TSHR-driven fibrosis and IL-6/STAT3 signaling. However, our Ad-TSHR-induced mouse model effectively simulates the chronic inflammatory and fibrotic aspects of TAO, it is important to acknowledge its limitations in fully replicating the intricate human orbital anatomy and the complete spectrum of human immune responses. These differences might influence the direct translatability of certain findings. Moreover, the use of orbital injection for microsphere delivery presents some challenges, as it is not yet a standard clinical practice for TAO treatment. Potential risks of orbital injections include the need for precise targeting and the possibility of adverse effects associated with direct injection into the orbital region. As such, future studies should focus on validating the safety and feasibility of orbital injection in human patients with TAO. Alternative delivery strategies, such as localized or systemic drug delivery systems, sub-Tenon injections, intravenous administration, or even advanced topical formulations could also enhance the therapeutic outcomes of CD34 Apt-modified MS, and their exploration will be crucial for clinical translation.

Toc is a monoclonal antibody targeting IL-6 receptors that has shown effectiveness in patients with TAO unresponsive to steroids,^7^ its therapeutic efficacy is often limited by challenges in targeted delivery. Previous research has explored various strategies for inhibiting IL-6 signaling in fibrotic diseases such as idiopathic pulmonary fibrosis and peritoneal fibrosis.^53^^,^^54^ However, our study distinguishes itself by uniquely combining PLGA-based microspheres with CD34-specific aptamers for the targeted delivery of Toc to CD34^+^ orbital fibroblasts in TAO. Apts have been used to capture various target molecules, including cells, proteins, and small molecules.^15^^,^^16^ Although Apt-guided delivery has been investigated in other contexts, such as TSHR-targeting aptamers for assessing TAO clinical activity^25^ and CD40-targeting RNA aptamers for TAO treatment,^55^ its application specifically targeting CD34^+^ orbital fibroblasts for sustained IL-6R inhibition in TAO via biodegradable microspheres represents a novel therapeutic strategy. This approach aims to enhance therapeutic efficacy by concentrating the drug at the pathological site, thereby minimizing systemic side effects and improving clinical outcomes by attenuating inflammation and fibrosis.

In conclusion, these findings affirm the therapeutic potential of CD34 Apt-coupled Toc/anti-IL-6R microspheres in alleviating inflammation and mitigating fibrotic tissue remodeling in the TAO orbital fibroblasts and the TAO mice model. The targeted delivery addresses local ocular manifestations by reducing inflammation and fibrosis and impacts systemic pathways, such as STAT3 signaling, offering a comprehensive strategy for managing TAO. Further investigation into delivery methods and the long-term effects of this treatment approach will be essential to translate these promising results into clinical practice.
